# Environmental Tobacco Smoke Exposure and Children’s Intelligence at 8–11 Years of Age

**DOI:** 10.1289/ehp.1307088

**Published:** 2014-06-03

**Authors:** Subin Park, Soo-Churl Cho, Yun-Chul Hong, Jae-Won Kim, Min-Sup Shin, Hee Jeong Yoo, Doug Hyun Han, Jae Hoon Cheong, Bung-Nyun Kim

**Affiliations:** 1 Department of Psychiatry, Seoul National Hospital, Seoul, Republic of Korea; 2 Division of Child and Adolescent Psychiatry, Department of Psychiatry, Seoul National University College of Medicine, Seoul, Republic of Korea; 3 Department of Preventive Medicine, an; 4 Institute of Environmental Medicine, Seoul National University College of Medicine, Seoul, Republic of Korea; 5 Department of Psychiatry, Chung Ang University, College of Medicine, Seoul, Republic of Korea; 6 Uimyung Research Institute for Neuroscience, Sahmyook University, Seoul, Republic of Korea

## Abstract

Background: Evidence supporting a link between postnatal environmental tobacco smoke (ETS) exposure and cognitive problems among children is mounting, but inconsistent.

Objectives: We examined the relationship between ETS exposure, measured using urine cotinine, and IQ scores in Korean school-aged children.

Methods: The participants were 996 children 8–11 years of age recruited from five administrative regions in South Korea. We performed a cross-sectional analysis of urinary cotinine concentrations and IQ scores obtained using the abbreviated form of a Korean version of the Wechsler Intelligence Scales for Children. Associations were adjusted for potential confounders, and estimates were derived with and without adjustment for mother’s Full-Scale IQ (FSIQ) score.

Results: After adjusting for sociodemographic and developmental covariates, urinary cotinine concentrations were inversely associated with FSIQ, Verbal IQ (VIQ), Performance IQ (PIQ), vocabulary, math, and block design scores. Following further adjustment for maternal IQ, only the VIQ scores remained significantly associated with urinary cotinine concentration (B = –0.31; 95% CI: –0.60, –0.03 for a 1-unit increase in natural log-transformed urine cotinine concentration; *p* = 0.03).

Conclusion: Urine cotinine concentrations were inversely associated with children’s VIQ scores before and after adjusting for maternal IQ. Further prospective studies with serial measurements of cotinine are needed to confirm our findings.

Citation: Park S, Cho SC, Hong YC, Kim JW, Shin MS, Yoo HJ, Han DH, Cheong JH, Kim BN. 2014. Environmental tobacco smoke exposure and children’s intelligence at 8–11 years of age. Environ Health Perspect 122:1123–1128; http://dx.doi.org/10.1289/ehp.1307088

## Introduction

Evidence supporting a link between postnatal environmental tobacco smoke (ETS) (secondhand smoke) exposure and cognitive and behavioral problems such as early grade retention (Byrd and Weitzman 1994), attention problems (Kollins et al. 2009; Twardella et al. 2010; Xu et al. 2010), and cognitive and intellectual deficits (Bauman et al. 1991; Eskenazi and Bergmann 1995; Johnson et al. 1999; Julvez et al. 2007; Yolton et al. 2005) among children is mounting, but inconsistent.

Various methodological limitations in previous studies have contributed to the lack of clarity in the findings. Previous research on the effects of tobacco smoke on children has been limited by reliance on parental reports of their children’s exposure, which may be limited by poor recall and may not include information on the extent of their children’s exposure to environmental tobacco smoke (Matt et al. 2000). Distinguishing between the effects of pre- and postnatal tobacco smoke exposure may be difficult because children who have been exposed prenatally tend also to be exposed postnatally (Knopik 2009). Moreover, a child’s cognitive functioning may be influenced by genetic, familial, educational, and social factors as well as prenatal (e.g., prenatal alcohol exposure and low birth weight), developmental (e.g., breastfeeding), and physical (e.g., neurological illness) factors (Bellinger 2000, 2004, 2008). These factors are likely to confound observed associations between the exposure of interest and the outcome (Mink et al. 2004). In particular, maternal IQ has been reported to be the single greatest predictor of child IQ in population-based studies, and is a well-known confounder of associations between children’s intellectual ability and other factors (Mink et al. 2004; Polderman et al. 2010). Persons with lower IQ scores in childhood have an increased risk of smoking (Gale et al. 2009; Kubicka et al. 2001; Martin et al. 2004). Given also the moderately high parent–offspring IQ correlation (Mascie-Taylor 1985; Reed and Rich 1982), parental IQ should also be considered as a potentially important candidate confounder in studies linking parental smoking with offspring IQ. Furthermore, maternal mental ability is related to knowledge and attitudes about the potentially harmful effects of secondhand smoking (Batty et al. 2006). Thus, further studies using reliable measures of ETS exposure and systematic investigation of confounding factors, including maternal IQ, are necessary to determine whether ETS is inversely associated with children’s cognition, independent of potential confounders.

Cotinine, the major metabolite of nicotine, is a biomarker for ETS exposure (Puig et al. 2008). We previously reported an association between urinary cotinine concentration and continuous performance test variables and attention-deficit/hyperactivity disorder (ADHD) and learning disability in Korean children (Cho et al. 2013). In the present study we examined the relationship between ETS exposure measured using urine cotinine concentrations and IQ in Korean children 8–11 years of age, with careful consideration of possible confounding factors including maternal IQ. We hypothesized that urinary cotinine levels would be associated with children’s IQ, independent of maternal IQ.

## Material and Methods

*Participants*. The present study comprised the second and third years of a 3-year research project, “Effects of pollution on neurobehavioral development, and future policies to protect our children,” funded by the Korean Ministry of Environment’s Eco-Technopia 21 Project. Participants were recruited from five administrative regions in Korea: Seoul and Seongnam are urban districts, Incheon and Ulsan are industrial cities, and Yeoncheon is a rural district. We selected 13 schools (two to three with the most representative demographic characteristics of each district) and sent parents of third- and fourth-grade children (age range, 8–11 years, *n* = 1,712) letters inviting them to participate in our study. The parents and children were given detailed information about the study, and we obtained written informed consent from both parents and children before any child was enrolled in the study. Of the 1,712 subjects initially contacted, 1,089 (response rate, 63.6%) agreed to participate. The study protocol was approved by the Institutional Review Board of Seoul National University Hospital.

The parents completed an extensive questionnaire comprising sociodemographic and relevant information concerning the children, including questions about socioeconomic status, paternal education, alcohol and tobacco use by the mothers during pregnancy, secondhand smoking status of the children, and medical, obstetrical, and developmental histories of the children. Among mothers who smoked during pregnancy, the number of cigarettes smoked per day and the pregnancy stage and duration of smoking was assessed. Secondhand smoking status was determined by asking the following question of the parents: “Does your child live in a household with a smoker?” Participants who responded positively were allocated to the “secondhand smoke exposure” group. Among participants exposed to secondhand smoke, the number of family members who smoked, the total number of cigarettes smoked by family members per day, and the degree of tobacco fumes in the home (almost none, little, moderate, or dense) were assessed.

*Measurement of urine cotinine levels*. The spot urine sample was collected in a 50-mL sterile specimen container at the school in the morning for each child and was refrigerated (2–8°C) immediately. Refrigerated specimens were transported to the laboratory within 2 hr. Cotinine direct ELISA kits (BioQuant, San Diego, CA, USA) were used to measure each child’s urine cotinine. Urine was diluted 1:100, and 10-μL samples were aliquoted in duplicate into 96-well microtiter plates. The urine was then incubated with 100 μL of the enzyme conjugate at room temperature for 60 min. We washed the wells with 300 μL distilled water and added 100 μL of the substrate to each well. The substrate was incubated at room temperature for 30 min, and the sample absorbance was measured using dual wavelength of 450 nm using a Versamax Microplate Reader (Molecular Device, Sunnyvale, CA, USA). The limit of detection (LOD) using this method was 1.0 ng/dL. For values below the LOD, we used half of the detection limit (LOD/2) in our calculations. The coefficients of variation (CVs) were 5.8–14.7% for interassay and 4.2–8.4% for intra-assay at environmental exposure levels, based on data from the manufacturer. Urine cotinine values that have not been corrected for urine creatinine values have been reported to be more highly correlated with parental smoking than creatinine-corrected values (Jatlow et al. 2003). Thus, we used creatinine-unadjusted urine cotinine values in our analyses.

*Assessment of the children’s IQ*. A trained examiner, blinded to the children’s cotinine levels, administered the IQ tests individually to each child in a quiet room. A licensed specialist in clinical psychology (M.S.S.) coordinated the tests and supervised the examiners. Details of the training process for the examiners have been described previously (Cho et al. 2010).

The children were administered the abbreviated form of the Korean Educational Development Institute’s Wechsler Intelligence Scales for Children (KEDI-WISC) (Park et al. 1996), which consists of two Verbal subtests, vocabulary and arithmetic, and two Performance subtests, picture arrangement and block design. Scaled scores for each subtest were computed and added to yield the sum of the scaled scores. Verbal IQ (VIQ) was the sum of scaled vocabulary and arithmetic subscale scores, Performance IQ (PIQ) was the sum of scaled picture arrangement and block design scores, and Full-Scale IQ (FSIQ) was the sum of all four subscale scores.

Kim and Kim (1986) reported that the correlations between the abbreviated and full version of the KEDI-WISC ranged from 0.89 to 0.92 on the four subtests; and the correlations between the two forms for VIQ, PIQ, and FSIQ were 0.97, 0.96, and 0.98, respectively, in Korean children 6–15 years of age.

*Assessment of the mothers’ IQ*. Each mother completed the short form of the Korean Wechsler Adult Intelligence Scale (K-WAIS), which has vocabulary, arithmetic, picture arrangement, and block design subtests, under the guidance of a trained examiner who was blinded to the children’s IQs. Lim et al. (2000) reported that the correlations between the abbreviated and full version of the K-WAIS for VIQ, PIQ, and FSIQ were 0.96, 0.96, and 0.97, respectively, in Koreans 18–53 years of age (mean ± SD, 35.90 ± 8.01 years).

*Statistical analysis*. We compared demographic and clinical characteristics between the children included and those excluded from the study using an independent *t*-test for continuous variables and a chi-square or Fisher’s exact test for categorical variables. We also compared urine cotinine concentrations between children with and without indirect smoking exposure by parental report and conducted Pearson correlation analysis between urine cotinine concentrations and the levels of ETS exposure by parental report.

To estimate associations between urine cotinine concentration and IQ scores, we performed linear regression analyses in male and female populations, separately, as well as in the total population. Urine cotinine concentrations were natural log (ln) transformed to achieve a normal distribution, and modeled as a continuous variable. In the regression analyses, the IQ score was the primary dependent variable, and concurrently measured urine cotinine concentration was the primary independent variable. Regression analyses were performed using a set of covariates based on established predictors of children’s cognitive function. We adjusted all models for the following developmental, socioeconomic, and familial influences on IQ: age (continuous, in years), sex, birth weight (continuous), history of breastfeeding (none vs. any), residential area [urban (Seoul and Seongnam), industrial (Incheon and Ulsan), or rural (Yeoncheon)], yearly family income (≥ US$25,000 vs. < US$25,000), and paternal educational years (continuous). The categorical variable (i.e., sex, history of breastfeeding, residential area, and yearly family income) was incorporated in the linear regression analyses using dummy variable coding. Because maternal IQ was expected to have the largest impact on the association between urine cotinine levels and children’s IQ (Polderman et al. 2010), we tested models with and without adjusting for maternal FSIQ. Furthermore, using an analysis of variance (ANOVA) and analysis of covariance (ANCOVA), we compared the children’s intelligence scores stratified by cotinine levels [nonexposed (< 1 ng/mL), low (1–14 ng/mL), medium (14–50 ng/mL), or high (> 50 ng/mL) exposure] according to urine cotinine categories used previously (Puig et al. 2008). In a previous study, Puig et al. (2008) reported that in the absence of established cut-offs for urinary cotinine to differentiate levels of ETS exposure in childhood, it was decided to apply the same stratification criteria as used for cord blood (Pichini et al. 2000). These cord blood cotinine level groups have been encountered in the international literature used to distinguish newborns of smoking mothers from newborns of nonsmoking mothers, and also to indicate passive exposure in newborns of non-smokers in related to the self-reported questionnaire (Bearer et al. 1997; Nafstad et al. 1996; Pichini et al. 2000). We also compared the geometric mean concentrations of urine cotinine among children with IQs < 85, 85–115, and > 115 using ANOVA and ANCOVA. The ANCOVA model 1 was adjusted for age, sex, birth weight, history of breastfeeding, residential area, early family income, and paternal educational level, and the ANCOVA model 2 was adjusted for the model 1 variables plus maternal IQ.

All statistical tests were conducted using the Statistical Package for the Social Sciences, version 19.0 (SPSS Inc., IBM, Chicago, IL, USA), and *p*-values < 0.05 were deemed statistically significant.

## Results

Initially, 1,089 children agreed to participate in the study. Of the 1,089 children, 1,007 (92.4%) produced sufficient urine to measure cotinine, and the remaining 82 were excluded from the study. An additional six participants were excluded for the following: history of seizure disorders (*n* = 2), history of neonatal hypoxia (*n* = 1), history of head trauma accompanied by cerebral hemorrhage (*n* = 1), and no IQ score available (*n* = 2).

Furthermore, five participants who had been exposed to maternal smoking during pregnancy were excluded to rule out the influence of maternal active smoking on outcomes, but we did not exclude participants if another household member had smoked during the pregnancy. Thus, a total of 996 subjects (with mean ages ranging from 8 to 11, and 524 males) were included in the statistical analysis (Table 1). The geographic distribution of the participants was as follows: 434 (43.6%) were from the two urban districts, 392 (39.3%) were from the two industrial cities, and 170 (17.1%) were from the rural district. A comparison of the demographic characteristics of participants included in and excluded from the study is shown in Table 1. Mean IQ and paternal educational years were significantly higher in children who were included compared to children who were excluded, and alcohol use during pregnancy was significantly less common in the included children. The geometric mean concentration of urine cotinine was 1.86 ng/dL [geometric standard deviation (GSD) = 3.53] for children living in urban districts, 1.79 ng/dL (GSD = 3.67) in industrial districts, and 1.82 ng/dL (GSD = 3.53) in the rural district (*p* = 0.880 for differences by residential area). Children with secondhand smoke exposure by parental report had significantly higher geometric mean urine cotinine concentrations than children without secondhand smoke exposure [2.42 ng/dL (GSD = 3.71) vs. 1.10 ng/dL (GSD = 2.69), *t* = 10.04, *p* < 0.001]. Urinary cotinine concentrations were positively and significantly correlated with the degree of tobacco fumes in the home by parental report (*r* = 0.24, *p* < 0.001), and were positively though not significantly correlated with the total number of cigarettes smoked by family members per day (*r* = 0.08, *p* = 0.077).

**Table 1:** Demographic characteristics of participants included and excluded from the study.

Characteristic	Children included (*n*= 996)	Children excluded (*n *= 93)^*a*^	*t*/*X*^2^	*p*-Value
Age (years)	9.1 ± 0.7	9.1 ± 0.7	–1.40	0.161
Sex (% female)	47.4	49.5	0.15	0.702
Child IQ	110.2 ± 14.3	104.4 ± 13.7	3.65	< 0.001
Paternal education (years)	13.8 ± 2.2	13.1 ± 2.3	2.58	0.010
Yearly income ≥ US$25,000	62.3	60.8	0.06	0.805
Maternal IQ	107.5 ± 11.6	106.8 ± 10.9	0.40	0.688
Alcohol use during pregnancy (%)	3.3	8.6	6.57	0.010
Child’s birth weight (kg)	3.2 ± 0.5	3.2 ± 0.4	0.61	0.540
History of breastfeeding (%)	59.7	49.3	2.92	0.087
Current SHS exposure by parental report (%)	57.5	58.9	0.05	0.817
No. of family members who smoked (among children with SHS exposure)	1.1 ± 0.2	1.2 ± 0.4	–1.86	0.069
Total no. of cigarettes smoked by family members per day (among children with SHS exposure)	45.4 ± 57.2	50.8 ± 57.6	–0.57	0.567
Degree of tobacco fumes in the home (among children with SHS exposure) (%)			1.79	0.618
Almost none	68.6	72.1
Little	21.9	16.3
Moderate	8.2	11.6
Dense	1.3	0
SHS, secondhand smoke. Values are mean ± SD unless otherwise specified. ^***a***^Children with a history of seizure disorders (*n *= 2), neonatal hypoxia (*n *= 1), or head trauma accompanied by cerebral hemorrhage (*n *= 1), urine output insufficient to measure cotinine (*n *= 82), no IQ score available (*n *= 2), or who had been exposed to maternal smoking during pregnancy (*n *= 5) were excluded from the main analyses.				

Table 2 shows estimated associations between urinary cotinine concentrations and IQ scores. After adjusting for sociodemographic and developmental covariates (model 1), urinary cotinine levels were significantly inversely associated with FSIQ, VIQ, PIQ, and all subtest scores except for the picture arrangement scores. Following further adjustment for maternal IQ (model 2), urinary cotinine levels showed a significant inverse association with VIQ scores (B = –0.31; 95% CI: –0.60, –0.03 for a 1-unit increase in ln-transformed urine cotinine concentration; *p* = 0.03), and an inverse but not statistically significant association with FSIQ (B = –0.56; 95% CI: –1.30, 0.17 for a 1-unit increase in ln-transformed urine cotinine concentration), math (B = –0.14; 95% CI: –0.29, 0.02 for a 1-unit increase in ln-transformed urine cotinine concentration), vocabulary (B = –0.13; 95% CI: –0.31, 0.05 for a 1-unit increase in ln-transformed urine cotinine concentration), and block design (B = –0.17; 95% CI: –0.35, 0.01 for a 1-unit increase in ln-transformed urine cotinine concentration) scores.

**Table 2:** Associations [B coefficients (95% CIs)] between ln-transformed urine cotinine concentration and IQ test scores among children 8–11 years of age.

Outcome	All (*n* = 996)				Boys (*n* = 524)				Girls (*n* = 472)			
	Model 1	*p*-Value	Model 2	*p*-Value	Model 1	*p*-Value	Model 2	*p*-Value	Model 1	*p*-Value	Model 2	*p*-Value
FSIQ	–1.26 (–1.98, –0.05)	0.001	–0.56 (–1.30, 0.17)	0.129	–1.10 (–2.10, 0.10)	0.027	–0.72 (–1.69, 0.25)	0.141	–1.18 (–2.30, –0.06)	0.035	0.04 (–1.14, 1.22)	0.942
VIQ	–0.50 (–0.78, –0.23)	< 0.001	–0.31 (–0.60, –0.03)	0.032	–0.45 (–0.80, –0.10)	0.014	–0.33 (–0.69, 0.03)	0.067	–0.51 (–0.97, –0.05)	0.029	–0.18 (–0.69, 0.33)	0.491
PIQ	–0.37 (–0.64, –0.10)	0.008	–0.14 (–0.43, 0.15)	0.337	–0.34 (–0.70, 0.01)	0.058	–0.24 (–0.62, 0.14)	0.198	–0.27 (–0.73, 0.19)	0.234	0.15 (–0.35, 0.65)	0.542
Math	–0.20 (–0.35, –0.06)	0.007	–0.14 (–0.29, 0.02)	0.081	–0.27 (–0.47, –0.07)	0.008	–0.21 (–0.40, –0.01)	0.037	–0.11 (–0.35, 0.13)	0.360	0.01 (–0.25, 0.26)	0.929
Vocabulary	–0.25 (–0.43, –0.08)	0.004	–0.13 (–0.31, 0.05)	0.163	–0.14 (–0.35, 0.10)	0.226	–0.08 (–0.32, 0.16)	0.520	–0.23 (–0.45, –0.01)	0.014	–0.12 (–0.41, 0.18)	0.412
Block design	–0.32 (–0.49, –0.15)	< 0.001	–0.17 (–0.35, 0.01)	0.059	–0.28 (–0.51, –0.04)	0.015	–0.19 (–0.42, 0.05)	0.112	–0.30 (–0.56, –0.04)	0.024	–0.06 (–0.35, 0.23)	0.663
Picture arrangement	0.04 (–0.13, 0.21)	0.666	0.12 (–0.06, 0.30)	0.186	0.04 (–0.18, 0.27)	0.745	0.05 (–0.19, 0.29)	0.662	0.10 (–0.16, 0.35)	0.477	0.28 (–0.02, 0.57)	0.062
Associations (B coefficients) are with 1-unit increase in ln-transformed urine cotinine concentrations. We used the Korean Educational Development Institute’s Wechsler Intelligence Scales for Children to measure outcomes. VIQ was the sum of scaled vocabulary and arithmetic subscale scores, PIQ was the sum of scaled picture arrangement and block design scores, and FSIQ was the sum of all four subscale scores. Model 1 was adjusted for age, sex, birth weight, history of breastfeeding, residential area, yearly family income, and paternal education level. Model 2 was adjusted as for model 1 plus maternal IQ (using the same subset).												

When we conducted analyses in male and female populations separately, urinary cotinine levels were significantly inversely associated with FSIQ, VIQ, and block design scores in both male and female populations after adjusting for sociodemographic and developmental covariates (model 1). In addition, urinary cotinine levels were significantly inversely associated with math scores in boys and with vocabulary scores in girls in model 1. In boys, urinary cotinine levels showed a significant inverse association with math scores (B = –0.21; 95% CI: –0.40, –0.01 for a 1-unit increase in ln-transformed urine cotinine concentration; *p* = 0.037) and an inverse, but not statistically significant associations with FSIQ (B = –0.72; 95% CI: –1.69, 0.25 for a 1-unit increase in ln-transformed urine cotinine concentration), VIQ (B = –0.33; 95% CI: –0.69, 0.03 for a 1-unit increase in ln-transformed urine cotinine concentration), PIQ (B = –0.24; 95% CI: –0.62, 0.14 for a 1-unit increase in ln-transformed urine cotinine concentration), and block design (B = –0.19; 95% CI: –0.42, 0.05 for a 1-unit increase in ln-transformed urine cotinine concentration) scores even after adjustment for maternal IQ (model 2). In contrast, in girls, associations between urinary cotinine levels and each IQ scores became null after adjustment for maternal IQ (Table 2).

**Figure 1:**
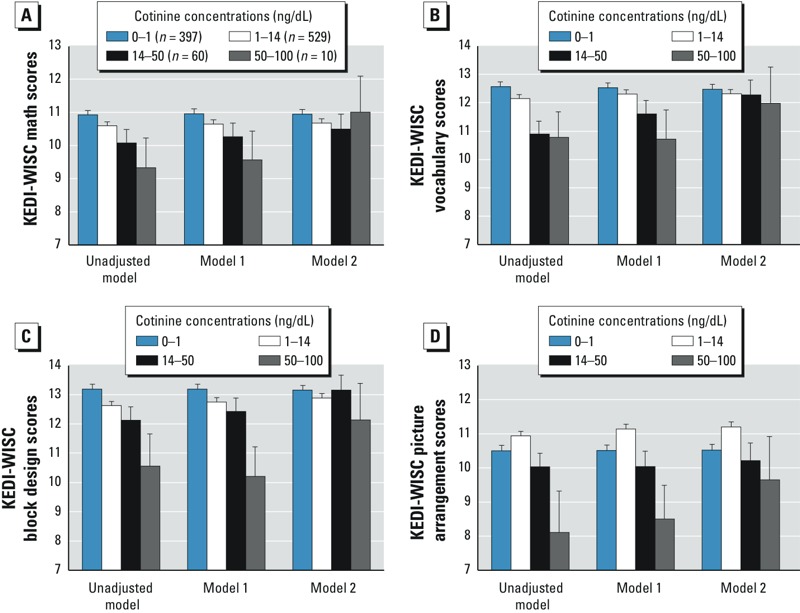
Mean math (*A*), vocabulary (*B*), block design (*C*), and picture arrangement (*D*) scores on the Korean Educational Development Institute’s Wechsler Intelligence Scales for Children (KEDI-WISC) estimated by urine cotinine concentrations. Model 1 was adjusted for age, sex, birth weight, history of breastfeeding, residential area, yearly family income, and paternal education level. Model 2 was adjusted as for model 1 plus maternal IQ (using the same subset). Error bars indicate standard errors.

Figure 1 shows unadjusted and adjusted estimates of each subtest scores by categorical cotinine levels. In unadjusted ANOVA model, math scores, vocabulary scores, and block design scores decreased as urine cotinine levels increased, and overall differences among the cotinine groups were significant [*F* = 2.83, degrees of freedom (df) = 3, *p* = 0.037; *F* = 5.28, df = 3, *p* = 0.001; and *F* = 4.74, df = 3, *p* = 0.003, respectively]. With regard to picture arrangement scores, scores were slightly lower in nonexposed children (urine cotinine levels < 1 ng/mL) than in low-exposed children (cotinine 1–14 ng/mL), but scores were progressively lower in the medium and high exposure groups, and overall differences among the groups were significant (*F* = 4.54, df = 3, *p* = 0.004). These trends were preserved even after adjustment for sociodemographic and developmental covariates (ANCOVA model 1), but did not remain after further adjustment for maternal IQ scores (ANCOVA model 2).

**Figure 2:**
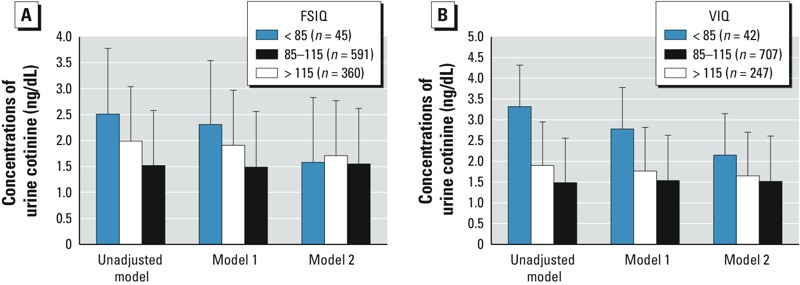
Geometric mean concentrations of urine cotinine according to FSIQ (*A*) and VIQ (*B*) on the abbreviated form of the Korean Educational Development Institute’s Wechsler Intelligence Scales for Children (KEDI-WISC). Model 1 was adjusted for age, sex, birth weight, history of breastfeeding, residential area, yearly family income, and paternal education level. Model 2 was adjusted as for model 1 plus maternal IQ (using the same subset). Error bars are standard errors.

Figure 2 shows unadjusted and adjusted estimates of urine cotinine levels according to the children’s FSIQ and VIQ. In unadjusted ANOVA model, urine cotinine levels decreased as FSIQ or VIQ scores increased, and overall differences among the IQ groups were significant (*F* = 6.47, df = 2, *p* = 0.002 for FSIQ and *F* = 8.05, df = 2, *p* < 0.001 for VIQ). For both FSIQ and VIQ, these trends were preserved even after adjustment for sociodemographic and developmental covariates (ANCOVA model 1). However, after further adjustment for maternal IQ scores, urine cotinine levels were slightly higher in mid-FSIQ group (FSIQ 85–115) than the lowest FSIQ group (FSIQ < 85), and urine cotinine levels did not differ among the three IQ ranges (*F* = 0.95, *p* = 0.386 for FSIQ, and *F* = 0.98, *p* = 0.375 for VIQ).

## Discussion

To our knowledge, this is the first investigation of the association between *in vivo* cotinine levels and IQ scores in children that takes maternal IQ scores and various sociodemographic and developmental confounders into consideration.

Yolton et al. (2005) administered two subtests of the Wide Range Achievement Test, the reading subtest and the math subtest, and two subtests from the Wechsler Intelligence Scale for Children (WISC-III) (Wechsler 1991), the block design subtest and the digit span subtest to the children and adolescents 6–16 years of age in the United States. They found an inverse association between serum cotinine concentration and reading scores, math scores, and block design scores. However, because maternal IQ, a well-known predictor of children’s IQ (Mink et al. 2004), was not measured, the possibility that the observed cognitive deficits were related to parental intellectual ability rather than ETS exposure could not be ruled out. Furthermore, maternal IQ may affect both exposure to ETS and the child’s IQ. Thus, to dissociate the association between maternal IQ and children’s urine cotinine, we estimated associations between children’s IQ scores and cotinine concentration after adjusting for maternal IQ.

Before adjusting for maternal IQ, we found a significant inverse association between urine cotinine concentration and the children’s FSIQ, VIQ, PIQ, vocabulary, math, and block design scores. These findings are consistent with those of Yolton et al. (2005) and other previous studies that used parental reports of children’s ETS exposure (Bauman et al. 1991; Byrd and Weitzman 1994; Eskenazi and Bergmann 1995; Johnson et al. 1999). However, after adjusting for maternal IQ, only VIQ remained significantly inversely associated with urine cotinine concentration. Our results suggest that previous studies that did not adjust for maternal IQ may have overestimated the association of postnatal ETS exposure with children’s cognitive ability. However, the confounding effect of maternal IQ on the association between cotinine concentrations and children’s VIQ was not substantial. In the previous study by Yolton et al. (2005), among the four cognitive subtests—math, reading, block design, and digit span—only reading scores remained significantly inversely associated with urine cotinine concentrations after adjusting for prenatal data including prenatal tobacco smoke exposure, birth weight, and neonatal intensive care unit stay. Although a direct comparison between our study and the study by the Yolton et al. is not possible because of the use of different cognitive outcome measures, both studies support the idea that verbal function (i.e., VIQ in our study and reading ability in the previous study) is most highly associated with postnatal ETS exposure. We also found that the highest IQ group showed the lowest urine cotinine levels, and the lowest IQ group showed the highest urine cotinine levels.

Potential mechanisms underlying associations between ETS exposure and intellectual function are not known. Rodent studies suggest that postnatal nicotine exposure may affect synaptic function and brain development in a manner similar to prenatal exposure (Britton et al. 2007; Gospe et al. 1996; Slotkin et al. 2007). Rodent exposure models suggest that postnatal nicotine exposure during critical periods of development disrupts corticothalamic circuitry, resulting in long-lasting dysregulation of sensory information processing in the cortex (Heath and Picciotto 2009). Children are at particular risk of ETS exposure because childhood is a critical period of vulnerability for the developing nervous system (Rice and Barone 2000).

Another notable finding was the sex differences in the association between urine cotinine concentrations and IQ scores. Generally, associations between cotinine concentrations and children’s test scores were more pronounced in males than in females, except for vocabulary scores. The association between cotinine levels and math scores was stronger for boys than girls based on model 1, and preserved for boys but essentially null for girls based on model 2. The association between cotinine levels and vocabulary scores was stronger for girls than for boys in model 1, but comparable for boys and girls in model 2. However, it should be noted that we did not do any direct statistical tests of interactions by sex or differences between boys and girls.

To our knowledge, there has been no study to examine the association between secondhand smoke and children’s cognitive function by sex. However, previous studies point to sex differences in neurobehavioral outcomes after prenatal nicotine exposure (Jacobsen et al. 2007) or active smoking (Jacobsen et al. 2005). These studies suggest stronger associations between ETS exposure and cognitive outcomes in males, consistent with our findings. Mechanisms underlying potential sex differences have not been identified. However, Slotkin et al. (2007) showed that sex differences in cholinergic and serotonergic pathways underlying cognition and behaviors emerge gradually with the onset of puberty. This study implies that nicotine changes the trajectory of the development of neural pathways by interacting with the sex differences that normally emerge with the onset of puberty (Pauly and Slotkin 2008).

Our study has several limitations. First, the cross-sectional design does not allow us to establish the temporal relation between ETS exposure and intellectual function in children. Second, certain demographic characteristics differed significantly between the participants who were included in and excluded from the study; this may have affected the generalizability of our findings to the source population for the original study. Third, although participants who had been exposed to maternal active smoking during pregnancy were excluded to rule out the influence of prenatal maternal active smoking on outcomes, we had no data on prenatal ETS exposure by biomarker or questionnaire. In addition, the use of self-reported questionnaires may underestimate the prevalence of maternal smoking during pregnancy. In a previous survey of 1,090 pregnant women in Korea, the percentage of smoking revealed by self-reporting was 0.55%, similar to that of the present study, but the percentage revealed in the same study by urinary cotinine measurements was 3.03% (Jhun et al. 2010). Fourth, we did not assess paternal IQ and used paternal education as a surrogate for paternal IQ. However, it is unclear whether paternal education is an adequate surrogate, and further studies that investigate paternal IQ as well as maternal IQ are needed. Fifth, because of the lack of data on other exposures to developmental neurotoxicants, we could not exclude the possibility that the association between urine cotinine levels and VIQ could have been caused by shared correlation with exposures to other developmental neurotoxicants or interaction effects between nicotine and other neurotoxicants. Finally, it is possible that a single urine cotinine measurement is not sufficient for examining the level and severity of ETS exposure. It is not clear whether short-term exposure (i.e., urine cotinine, which reflects a nicotine exposure of 2–3 days) represents a child’s chronic exposure or indicates the short-term toxicity of ETS exposure. Thus, further studies using serial measurements of cotinine are needed to obtain a more accurate estimate of ETS exposure.

## Conclusion

In conclusion, the results of our study support previously observed inverse associations between ETS exposure and measures of intellectual function. Furthermore, urine cotinine levels were inversely associated with children’s VIQ, even after adjusting for mother’s IQ, in our study population.
